# Individual factors associated with the use of oral health services by adults according to sex: a structural equation modeling approach

**DOI:** 10.1590/0102-311XEN074025

**Published:** 2026-01-09

**Authors:** Nildelaine Cristina Costa, Jorge Alexandre Barbosa Neves, Luciano Mattar, Mauro Henrique Nogueira Guimarães Abreu, Renata de Castro Martins

**Affiliations:** 1 Universidade Federal de Minas Gerais, Belo Horizonte, Brasil.

**Keywords:** Dental Health Services, Dental Care, Sex, Serviços de Saúde Bucal, Assistência Odontológica, Sexo, Servicios de Salud Dental, Atención Odontológica, Sexo

## Abstract

This study evaluated individual factors associated with the use of oral health services by Brazilian adults according to sex. This cross-sectional analysis used secondary data from the 2019 *Brazilian National Health Survey*. Data from 65,803 adults aged 18-59 years were included. The outcome was “the use of oral health services”. Independent variables were grouped into three constructs: sociodemographic data as predisposing factors, economic and access-related characteristics as capacity factors, and data on oral health conditions as need factors. Data were analyzed in Stata 15.0, using the structural equation modeling. Structural models were estimated for males and females. Analysis stratified by sex presented adequate adjustment indicators: comparative fit index > 0.90, Tucker-Lewis index > 0.90, and standardized root mean square residual < 0.05. The final models showed that predisposing factors effected capacity factors (β = 0.61 for men; β = 0.54 for women), which, in turn, directly affected need factors (β = -0.24 for men; β = -0.26 for women) and the outcome (β = 0.23 for men; β = 1.15 for women). The final structural model demonstrated a good fit, with need factors also directly affecting the outcome (β = 0.10 for men; β = 0.09 for women). All coefficients were statistically significant (p < 0.001). The use of oral health services was indirectly influenced by sociodemographic factors, and directly and indirectly influenced by economic and access-related factors. Oral health conditions had a direct effect on service use, with no variation between sexes. However, men were more affected by predisposing and capacity factors than women.

## Abstract (pt)

Este estudo avaliou os fatores individuais associados à utilização de serviços de saúde bucal por adultos brasileiros segundo sexo. Este estudo transversal utilizou dados secundários da *Pesquisa Nacional de Saúde* de 2019. Foram utilizados dados de 65.803 adultos, com idade entre 18 e 59 anos. O desfecho foi “utilização de serviços de saúde bucal”. As variáveis ​​independentes foram agrupadas em três constructos: dados sociodemográficos como fatores predisponentes, dados econômicos e de acesso aos serviços como fatores de capacidade e dados sobre condições de saúde bucal como fatores de necessidade. Os dados foram analisados ​​no Stata 15.0, utilizando a modelagem de equações estruturais. Os modelos estruturais foram construídos com estimativas separadas para homens e mulheres. A análise estratificada por sexo apresentou indicadores de ajuste adequados: índice de ajuste comparativo > 0,90, índice de Tucker-Lewis > 0,90, raiz quadrática média residual padronizada < 0,05. Os modelos finais mostraram que o construto fatores predisponentes teve efeito sobre o construto fatores de capacidade (β = 0,61 para homens; β = 0,54 para mulheres), que, por sua vez, teve efeitos diretos sobre o construto fatores de necessidade (β = -0,24 para homens; β = -0,26 para mulheres) e sobre o desfecho (β = 0,23 para homens; β = 1,15 para mulheres). O modelo estrutural final ajustou-se bem ao efeito de fatores de necessidade sobre o desfecho (β = 0,10 para homens; β = 0,09 para mulheres), com todos os coeficientes sendo considerados significativos (p < 0,001). A utilização de serviços de saúde bucal foi influenciada indiretamente por fatores sociodemográficos bem como direta e indiretamente por fatores econômicos e de acesso. As condições de saúde bucal tiveram efeito direto sobre a utilização de serviços de saúde, sem variação entre os sexos. No entanto, os homens foram mais afetados por fatores predisponentes e de capacidade do que as mulheres.

## Abstract (es)

Este estudio evaluó los factores individuales asociados con el uso de los servicios de salud bucodental por parte de los adultos brasileños según el sexo. Se trata de un estudio transversal que utilizó datos secundarios de la *Encuesta Nacional de Salud* de 2019, con datos de 65.803 adultos de entre 18 y 59 años. El resultado fue “el uso de los servicios de salud bucodental”. Las variables independientes se agruparon en tres constructos: datos sociodemográficos como factores predisponentes, datos económicos y acceso a los servicios como factores de capacidad y datos sobre las condiciones de salud bucodental como factores de necesidad. Los datos se analizaron en Stata 15.0, aplicando el modelo de ecuaciones estructurales. Se construyeron modelos estructurales con estimaciones separadas para hombres y mujeres. El análisis estratificado por sexo presentó indicadores de ajuste adecuados: índice de ajuste comparativo > 0,90, índice de Tucker-Lewis > 0,90, raíz cuadrada media estandarizada residual < 0,05. Los modelos finales mostraron que el constructo factores predisponentes tenía un efecto sobre el constructo factores de capacidad (β = 0,61 para hombres; β = 0,54 para mujeres), que, a su vez, tenía efectos directos sobre el constructo factores de necesidad (β = -0,24 para hombres; β = -0,26 para mujeres) y en el resultado (β = 0,23 para hombres; β = 1,15 para mujeres). El modelo estructural final se ajustó bien con el efecto de los factores de necesidad sobre la variable de resultado (β = 0,10 para hombres; β = 0,09 para mujeres), y todos los coeficientes se consideraron significativos (p < 0,001). El uso de los servicios de salud bucodental se vio influido indirectamente por factores sociodemográficos y, directa e indirectamente, por factores económicos y de acceso. Las condiciones de salud bucodental tuvieron un efecto directo en el uso de los servicios de salud, sin variaciones entre los sexos. Sin embargo, los hombres se vieron más afectados por los factores predisponentes y los de capacidad que las mujeres.

## Introduction

The use of oral health services is fundamental in maintaining overall health and improving quality of life. Regular dental visits help prevent oral diseases, enable early diagnosis, and promote healthy habits ^1^. Globally, approximately 3.5 billion individuals suffer from oral diseases ^2^, highlighting the need to ensure universal access to oral health services without discrimination based on sex, ethnicity, or sociocultural background. However, despite global efforts to develop equitable healthcare systems, use of oral health services remains limited among socioeconomically vulnerable populations ^1^ ^,^ ^2^ ^,^ ^3^ ^,^ ^4^ ^,^ ^5^ and occurs unequally between sexes ^1^ ^,^ ^2^ ^,^ ^6^ ^,^ ^7^.

These differences reflect distinct approaches to oral health, encompassing the frequency of dental visits and attitudes toward both preventive and curative care ^6^ ^,^ ^8^ ^,^ ^9^ ^,^ ^10^ ^,^ ^11^. Generally, women tend to use these services more regularly, which is associated with greater awareness of the importance of oral health and the adoption of more consistent preventive behaviors, such as routine dental checkups ^1^ ^,^ ^6^ ^,^ ^9^ ^,^ ^10^. Socioeconomic, behavioral, and demographic factors exert distinct influences on men and women ^6^ ^,^ ^8^ ^,^ ^10^. Women with higher levels of education and income are more likely to use oral health services regularly ^6^ ^,^ ^10^, while men, regardless of socioeconomic status, are less inclined to seek preventive care and typically visit the dentist only in urgent situations ^6^ ^,^ ^9^ ^,^ ^11^. Cultural and social aspects, including masculinity norms, can also impact men’s adherence to treatment ^12^ ^,^ ^13^.

Although the literature acknowledges sex-based differences in oral health ^6^ ^,^ ^8^ ^,^ ^10^, no studies have adopted a sex-stratified approach to analyze how sociodemographic and health determinants directly and indirectly influence use of oral health services. Structural equation modeling (SEM) has been increasingly employed in health sciences to examine and test relationships that require the simultaneous analysis of multiple pathways ^14^. This study aimed to analyze the direct and indirect relationships between factors associated with the use of dental services by Brazilian adult men and women. It advances the understanding of sex differences by adopting an analytical approach that enables the simultaneous estimation of both direct and indirect effects of multiple determinants on use of oral health services, while also capturing the complex interactions among them ^14^ ^,^ ^15^. The findings may contribute to the development of strategies that address the specific needs of each sex, promoting more equitable and effective use of such services.

## Methods

This cross-sectional study used secondary data from the 2019 *Brazilian National Health Survey* (PNS, acronym in Portuguese). The 2019 PNS was reviewed and approved by the Brazilian National Research Ethics Commission of the Brazilian National Health Council (CAAE 11713319.7.0000.0008). This publicly available database can be accessed at https://www.ibge.gov.br/estatisticas/sociais/saude/9160-pesquisa-nacional-de-saude.html.

The 2019 PNS was conducted by the Brazilian Institute of Geography and Statistics (IBGE, acronym in Portuguese) in partnership with the Brazilian Ministry of Health. The survey enabled a detailed analysis of health conditions across different regions and socioeconomic contexts in the country ^16^ ^,^ ^17^.

The complex 3-stage sampling design was developed to obtain a representative sample of the Brazilian population. In the first stage, the primary sampling units (PSUs) corresponding to the census tracts were geographically selected and stratified. In the second stage, census tracts were selected by probability proportional to size, using the total number of households within each tract. Households were then selected by simple random sampling, ensuring equal selection probability for all households within each tract. In the third stage, one resident aged 15 or over was randomly selected from each household to complete the individual health questionnaire, also using simple random sampling ^16^ ^,^ ^17^. The final 2019 PNS sample included 108,225 interviewed households. However, the sample used in this study included data from 65,803 adults aged 18-59 years.

The dependent variable (use of oral health services) was based on the question: “When was your last visit to the dentist?”. The original response options (up to 1 year; more than 1 year to 2 years; more than 2 years to 3 years; more than 3 years; never went to the dentist) were dichotomized into “used” (up to 1 year; more than 1 year to 2 years; more than 2 years to 3 years; more than 3 years) or “not used” (never went to the dentist). Independent variables included sociodemographic and economic factors, access to health services, and oral health conditions.

Sociodemographic and economic variables included: type of census location (rural; urban), treated water supply (general distribution network; deep or artesian well; shallow, phreatic, or cacimba well; fountain or spring; stored rainwater; other) dichotomized into “yes” (general distribution network) and “no” (other options); sanitary sewage (general sewage or rainwater network; septic tank connected to the grid; septic tank not connected to the grid; rudimentary septic tank; ditch; river, lake, stream, or sea; other) dichotomized into “yes” (general sewage or rainwater network) and “no” (other options); garbage collection (collected directly by cleaning service; collected in a cleaning service bucket; burned on the property; buried on the property; disposed of on vacant land or public place; other) dichotomized into “yes” (collected directly by cleaning service) and “no” (other options); per capita income (up to 1/4 minimum wage; more than 1/4 to 1/2 minimum wage; more than 1/2 to 1 minimum wage; more than 1 to 2 minimum wages; more than 2 to 3 minimum wages; more than 3 to 5 minimum wages; more than 5 minimum wages), categorized into up to 1 minimum wage, above 1 to 3 minimum wages, above 3 minimum wages; paid work (“yes” or “no”); and internet access (“yes” or “no”).

Access to health services variables included private dental coverage (“yes” or “no”); registration in a basic health unit (BHU) (“yes”, “no”, or “do not know”), dichotomized into “yes” or “no” (no/do not know).

Finally, oral health condition variables included tooth loss in the maxilla (total loss; partial loss; no loss), dichotomized into “yes” (total loss/partial loss) and “no” (no loss); tooth loss in the mandible (total loss; partial loss; no loss), dichotomized into “yes” (total loss/partial loss) and “no” (no loss); use of dental prostheses (no need; does not use; does use), dichotomized into “no” (does not use/no need) and “yes” (does use); self-perceived oral health (very good; good; average; bad; very bad), dichotomized into “very good/good”, “average”, and “poor/very poor”.

Descriptive data analysis was performed to explore the distribution of variables using Stata 15.0 (https://www.stata.com). SEM, a useful technique for analyzing multiple independent variables, was used to evaluate the relationship between independent variables and the outcome ^14^ ^,^ ^15^. To account for the complex sampling design, the PSUs and “Stratum” variables were used for sample stratification, while the “Weight of resident selected with calibration” variable was applied for sample weighting. Models were estimated using the R software (http://www.r-project.org) with the *lavaan* ^18^ and *lavaan.survey* ^19^ packages, which are specifically developed for factor analysis and SEM. In SEM, variables are essential for representing complex interrelationships and are classified based on their function as latent variables or indicator variables ^14^ ^,^ ^15^. Latent variables, or constructs, represent theoretical concepts that cannot be directly observed and are indirectly measured via indicator variables, which are directly observed and collectively form latent constructs ^14^ ^,^ ^20^. SEM combines elements of factor analysis and multiple regression to examine both direct and indirect relationships among variables, enabling the decomposition of total effects within the model. The relationships between observable (indicator) and latent variables, as well as among latent constructs themselves, are analyzed using path diagrams or path analysis. In these diagrams, circles or ellipses denote latent variables, rectangles or squares represent indicator variables, and arrows indicate the direction and type of effect among them. Unidirectional arrows from latent to indicator variables show how constructs are measured, unidirectional arrows between latent variables indicate causal hypotheses, and bidirectional arrows represent correlations without specified causal direction ^14^ ^,^ ^15^.

**Figure 1:**
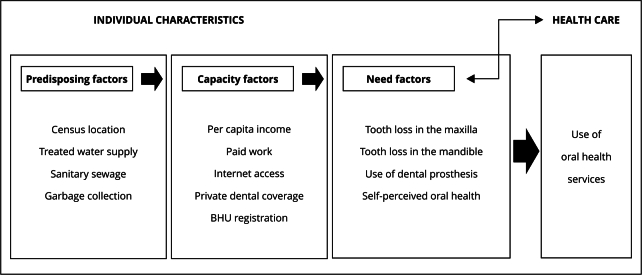
Flowchart, according to Andersen’s theoretical model ^21^, adapted to the research variables.

To use SEM, it is essential to have a theoretical model that justifies the inclusion of indicator variables ^14^ ^,^ ^15^ ^,^ ^20^. This study was based on Andersen’s theoretical model ^21^, which posits that the use of health services is influenced by three main groups of factors: (1) predisposing factors, such as sociodemographic characteristics; (2) capacity factors, such as economic conditions and access to health services; (3) and need factors, encompassing both self-perceived and professionally diagnosed oral health conditions. The interaction among these components shapes the use of health services. Predisposing factors affects capacity factors, which influences need factors, the most proximal factor and an important predictor of the use of health services ^21^ ^,^ ^22^. Variables included in each construct are shown in Figure 1.

BHU: basic health unit.

The decision to use dichotomous variables was based on previous population-based studies ^23^ ^,^ ^24^ ^,^ ^25^ that also employed this approach to examine health service utilization. Models were constructed using dichotomous and ordinal variables with polychoric correlation matrices. All analyses were performed using the weighted least squares mean and variance adjusted (WLSMV) estimator, which is appropriate for models with dichotomous observed variables ^26^. The modeling was conducted in two stages. First, the measurement model was tested using confirmatory factor analysis (CFA) to test latent variable indicators (observed variables) according to factor loadings and statistical significance. For standardized estimates used to construct latent variables predisposing factors, capacity factors, and need factors, factor loadings > 0.03 with a p < 0.05 were considered indicative of a moderately high relationship between the observed variable and the construct ^20^. In the second stage, structural models were evaluated for the total sample to estimate direct, indirect, and total effects.

## Results

Data from 31,469 (47.83%) adult men and 34,334 (52.17%) adult women were analyzed. Most adults included in this study reported having used oral health services (98.3%), lived in urban areas (86.4%), and had access to treated water supply (85.1%), sanitary sewage (65.4%), and garbage collection (91.6%). More than half of participants had a per capita household income of up to 1 minimum wage (53.8%), were employed in paid work (65.5%), and had internet access (89.1%). Moreover, most respondents did not have private dental insurance (85.5%) and were registered at a BHU (61.4%). Regarding oral health conditions, the majority reported tooth loss in both the maxilla (52.3%) and mandible (54.4%), did not use dental prosthesis (77.7%), and self-rated their oral health as very good/good (70.6%) (Table 1).

**Table 1:** Descriptive analysis and 90% confidence intervals (90%CI) of variables used according to sex. The 2019 *Brazilian National Health Survey*.

Variables	Brazil	Male	Female
% (90%CI)	% (90%CI)	% (90%CI)
Use of oral health services			
Not used	1.7 (1.6-1.9)	2.5 (2.3-2.8)	1.0 (0.9-1.2)
Used	98.3 (98.3-98.4)	97.5 (97.2-97.7)	99.0 (98.8-99.1)
Predisposing factors			
Census location			
Rural	13.6 (13.2-14.1)	15.2 (14.6-15.7)	12.2 (11.8-12.7)
Urban	86.4 (85.9-86.8)	84.8 (84.3-85.4)	87.8 (87.3-88.2)
Treated water supply			
No	14.9 (14.2-15.5)	16.2 (15.5-16.9)	13.7 (13.1-14.3)
Yes	85.1 (84.5-85.8)	83.8 (83.1-84.5)	86.3 (85.7-86.9)
Sanitary sewage			
No	34.6 (33.6-35.6)	35.7 (34.7-36.8)	33.5 (32.6-34.5)
Yes	65.4 (64.4-66.4)	64.3 (63.2-65.3)	66.5 (65.5-67.4)
Garbage collection			
No	8.4 (8.0-8.8)	9.6 (9.2-10.1)	7.3 (7.0-7.7)
Yes	91.6 (91.2-92.0)	90.4 (89.9-90.8)	92.7 (92.3-93.0)
Capacity factors			
Per capita income (minimum wages)			
Up to 1	53.8 (52.9-54.7)	51.2 (50.3-52.2)	56.2 (55.2-57.1)
Above 1 to 3	35.8 (35.0-36.6)	37.6 (36.7-38.5)	34.1 (33.3-35.0)
Above 3	10.4 (9.8-11.0)	11.2 (10.5-11.9)	9.7 (9.1-10.3)
Paid work			
No	34.5 (33.8-35.2)	23.4 (22.6-24.2)	44.7 (43.8-45.5)
Yes	65.5 (64.8-66.2)	76.6 (75.8-77.4)	55.3 (54.5-56.2)
Internet access			
No	10.9 (10.5-11.3)	11.9 (11.5-12.4)	10.0 (9.6-10.4)
Yes	89.1 (87.7-88.5)	88.1 (87.6-88.5)	90.0 (89.6-90.4)
Private dental insurance			
No	85.5 (84.9-86.0)	85.5 (84.9-86.2)	85.4 (84.8-86.0)
Yes	14.5 (14.0-15.1)	14.5 (13.8-15.1)	14.6 (14.0-15.2)
BHU registration			
No	38.6 (37.4-39.7)	39.1 (38.0-40.3)	38.0 (36.9-39.1)
Yes	61.4 (60.3-62.6)	60.9 (59.7-62.0)	62.0 (60.9-63.1)
Need factors			
Tooth loss in the maxilla			
No	47.7 (46.9-48.5)	50.7 (49.8-51.5)	45.0 (44.1-46.0)
Yes	52.3 (51.5-53.1)	49.3 (48.5-50.2)	55.0 (54.0-55.9)
Tooth loss in the mandible			
No	45.6 (44.8-46.4)	48.3 (47.4-49.2)	43.1 (42.2-44.0)
Yes	54.4 (53.6-55.2)	51.7 (50.8-52.6)	56.9 (56.0-57.8)
Use of dental prosthesis			
No	77.7 (77.1-78.3)	80.7 (79.9-81.4)	75.0 (74.3-75.6)
Yes	22.3 (21.7-22.9)	19.3 (18.6-20.1)	22.3 (21.8-22.8)
Self-perceived oral health			
Poor/Very poor	4.8 (4.5-5.1)	4.6 (4.2-4.9)	5.0 (4.6-5.5)
Average	24.6 (24.0-25.3)	25.9 (25.1-26.7)	23.5 (22.9-24.2)
Good/Very good	70.6 (69.9-71.2)	69.6 (68.7-70.4)	71.5 (70.7-72.2)

BHU: basic health unit.

Table 2 presents CFA results, including factor loadings and statistical significance of the observed variables comprising the latent constructs in the SEM for men and women. The CFA showed that all variables had statistically significant factor loadings (p < 0.0001), confirming their adequacy in representing latent constructs. Among predisposing factors, sanitary sewage had the highest loading for both men (β = 1.121) and women (β = 1.368), followed by access to treated water and garbage collection. Regarding capacity factors, paid employment and internet access stood out, while registration in BHUs showed a negative association. Among need factors, tooth loss and denture use had the highest loadings, while negative self-perception of oral health was inversely associated.

**Table 2:** Confirmatory factor analysis (CFA) with factor loadings and statistical significance of the observed variables of latent variables in the models according to sex. The 2019 *Brazilian National Health Survey*.

Variables	Male			Female		
	β	SE	p-value	β	SE	p-value
Predisposing factors						
Census location	1.000			1.000		
Treated water supply	0.885	0.011	< 0.0001	0.918	0.013	< 0.0001
Sanitary sewage	1.121	0.015	< 0.0001	1.368	0.021	< 0.0001
Garbage collection	0.732	0.009	< 0.0001	0.698	0.010	< 0.0001
Capacity factors						
Per capita income	1.000			1.000		
Paid work	0.237	0.006	< 0.0001	0.497	0.009	< 0.0001
Internet access	0.411	0.008	< 0.0001	0.305	0.006	< 0.0001
Private dental insurance	0.294	0.005	< 0.0001	0.295	0.005	< 0.0001
BHU registration	-0.404	0.008	< 0.0001	-0.421	0.008	< 0.0001
Need factors						
Tooth loss in the maxilla	1.000			1.000		
Tooth loss in the mandible	0.793	0.013	< 0.0001	0.829	0.012	< 0.0001
Use of dental prosthesis	0.469	0.008	< 0.0001	0.595	0.008	< 0.0001
Self-perceived oral health	-0.423	0.005	< 0.0001	-0.405	0.008	< 0.0001

BHU: basic health unit; SE: standard error.

The final structural models, which demonstrated excellent fit, were estimated separately for men and women. The following fit indices were considered good: (a) p > 0.05 for the chi-square test (χ^2^); (b) root mean square error of approximation (RMSEA) < 0.06 or < 0.08 with the upper limit of the 90% confidence interval (90%CI) < 0.10; (c) standardized root mean square residual (SRMR) < 0.08; and (d) comparative fit index (CFI) and Tucker-Lewis index (TLI) values > 0.90 ^14^ ^,^ ^15^ ^,^ ^20^. Table 3 shows model fit indices for men and women.

**Table 3:** Model fit indices according to sex. The 2019 *Brazilian National Health Survey*.

Fit indices	Male model	Female model
χ^2^	77,412.920	4,542.924
Degrees of freedom	91	74
p-value	< 0.0001	< 0.0001
RMSEA	0.041	0.042
90%CI	0.040-0.042	0.041-0.043
CFI	0.949	0.944
TLI	0.937	0.931
SRMR	0.043	0.048

90%CI: 90% confidence interval; CFI: comparative fit index; RMSEA: root mean square error of approximation; SRMR: standardized root mean square residual; TLI: Tucker-Lewis index.

Note: final model with best fit.

**Figure 2:**
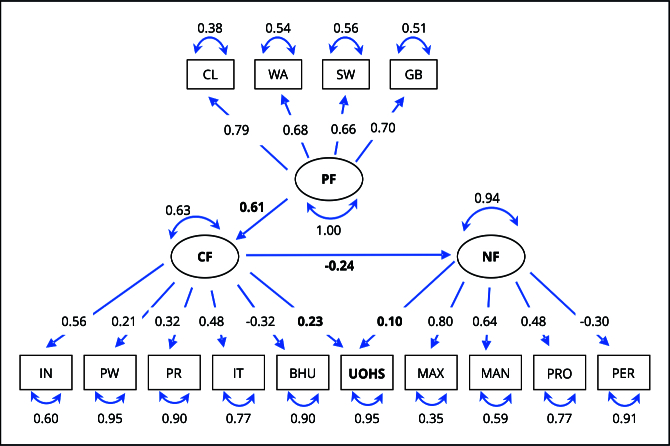
Final model for male, latent, and observed variables, and their respective adjusted factor loadings. The 2019 *Brazilian National Health Survey*.

BHU: basic health unit registration; CF: capacity factors; CL: census location; GB: garbage collection; IN: per capita income; IT: internet access; MAN: tooth loss in the mandible; MAX: tooth loss in the maxilla; NF: need factors; PER: self-perceived oral health; PF: predisposing factors; PR: private dental coverage; PRO: use of dental prosthesis; PW: paid work; SW: sanitary sewage; UOHS: use of oral health services; WA: treated water supply.

**Figure 3:**
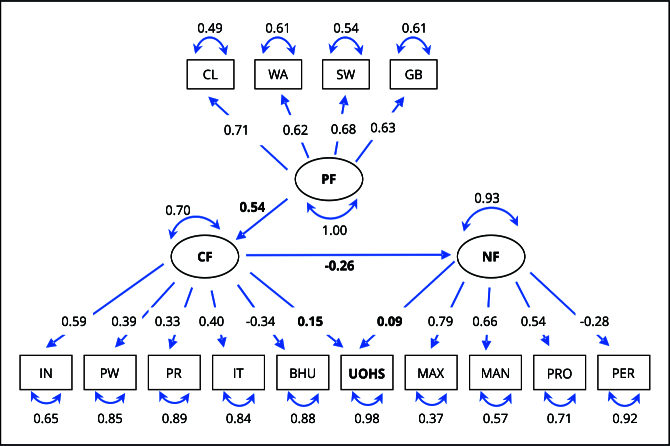
Final model for female, latent, and observed variables, and their respective adjusted factor loadings. The 2019 *Brazilian National Health Survey*.

BHU: basic health unit registration; CF: capacity factors; CL: census location; GB: garbage collection; IN: per capita income; IT: internet access; MAN: tooth loss in the mandible; MAX: tooth loss in the maxilla; NF: need factors; PER: self-perceived oral health; PF: predisposing factors; PR: private dental coverage; PRO: use of dental prosthesis; PW: paid work; SW: sanitary sewage; UOHS: use of oral health services; WA: treated water supply.

Figures 2 and 3 present the structural models with adjusted total impacts for men and women. They include the observed variables that formed the constructs and the direct and indirect relationships among predisposing, capacity, and need factors and use of oral health services.

Predisposing factors had a positive effect on capacity factors (β = 0.61 for men; β = 0.54 for women), which, in turn, had a direct effect on need factors (β = -0.24 for men; β = -0.26 for women) and on use of oral health services (β = 0.23 for men; β = 1.15 for women). The model also indicated a direct effect of need factors on use of oral health services (β = 0.10 for men; β = 0.09 for women), with all coefficients proving to be significant (p < 0.001) (Figures 2 and 3).

The observed variables with the highest factor loadings within the constructs were as follows: census location in predisposing factors (β = 0.79 for men; β = 0.71 for women); income in capacity factors (β = 0.56 for men; β = 0.59 for women); and tooth loss in the maxilla in need factors (β = 0.80 for men; β = 0.79 for women) (Figures 2 and 3).

## Discussion

Sociodemographic factors had an indirect effect on use of oral health services, while economic factors and access to services had both direct and indirect effects. Moreover, oral health conditions had a direct effect on use of oral health services.

Most adults lived in urban areas and had access to treated water, garbage collection, and the internet. However, reports on sanitary sewage were lower than 66%. These services are essential for public health, yet sanitary sewage coverage remains limited, reflecting persistent inequalities. The lack of adequate sewage infrastructure can compromise water quality, increase the risk of infectious diseases, and ultimately affect population health ^27^ ^,^ ^28^.

The adults surveyed reported having used oral health services at least once and presented moderate socioeconomic conditions, indicating good overall coverage of oral health services in Brazil. However, over 85% lacked private dental insurance, demonstrating a strong dependence on the Brazilian Unified National Health System (SUS, acronym in Portuguese). Although more than 60% of households are registered at SUS, studies show that most dental appointments in Brazil occur in the private sector ^3^ ^,^ ^17^ ^,^ ^29^, suggesting that many individuals pay out-of-pocket for dental care. It is also noteworthy that, although approximately 98% of participants reported having visited a dentist at least once, this does not necessarily indicate adherence to a regular preventive routine.

More than half of the surveyed adults reported tooth loss in both the maxilla and mandible, suggesting challenges in maintaining oral health, possibly associated with barriers in accessing oral health services ^23^ ^,^ ^30^ ^,^ ^31^. Nonetheless, the self-perceived good or very good oral health among most participants suggests that tooth loss may not significantly affect their oral health perception ^23^. This indicates a discrepancy between normative need, perceived need, and actual demand for services, which can be explained by adaptation to oral conditions, low priority given to aesthetics or oral function, and difficulties in accessing services ^23^ ^,^ ^30^ ^,^ ^32^ ^,^ ^33^.

The CFA results support the validity of Andersen’s behavioral model in explaining use of oral health services, demonstrating that predisposing, capacity, and need factors are significantly associated with the outcome ^21^ ^,^ ^22^. The greater influence of variables related to basic sanitation and oral health conditions reinforces the need to expand access to services in areas with limited infrastructure, as well as to strengthen preventive and rehabilitative oral health care, especially among vulnerable groups ^34^. Validation of the model for both sexes suggests theoretical consistency; however, the presence of gender inequalities should be considered.

SEM showed an indirect and positive effect of predisposing factors on use of oral health services, mediated by capacity factors. This suggests that individuals living in urban areas with adequate basic sanitation are more likely to use these services when they also have economic and access-related conditions ^21^ ^,^ ^22^. The moderate-to-high factor loadings (β = 0.61 for men and β = 0.54 for women) reinforce the statistical relevance of these associations ^15^. The positive association indicates that an increase in predisposing factors enhances individuals’ capacity to adhere to oral health services ^8^ ^,^ ^29^. The higher predisposing factors loading among men suggests a greater impact of sociodemographic conditions on use of oral health services in this group, consistent with studies associating being male and living in rural areas with lower service use ^7^ ^,^ ^8^. This lower use has been attributed to sociocultural barriers, including the undervaluation of oral health, masculinity norms, and prioritization of work ^1^ ^,^ ^35^ over self-care ^8^ ^,^ ^9^ ^,^ ^10^. Although limited access in remote areas affects both sexes, women tend to show greater motivation to overcome these barriers − a behavior historically linked to gender roles assigning them greater responsibility for healthcare, thereby encouraging help-seeking behaviors, including for oral health ^7^ ^,^ ^36^.

Capacity factors had an indirect and negative impact mediated by need factors and a direct and positive impact on use of oral health services for both genders. These findings are consistent with Andersen’s model, which posits that while individuals may have both predisposing and enabling conditions such as access and income, effective utilization of health services ultimately depends on the presence of perceived need ^21^ ^,^ ^22^. The indirect impact of capacity factors was similar for men and women. When analyzing factor loadings, the magnitude of the absolute value indicates the strength of the association, whereas the sign reveals its direction, with negative values denoting inverse relationships and positive values representing direct relationships ^14^ ^,^ ^15^ ^,^ ^20^ ^,^ ^26^. The negative factor loading shows that increasing capacity factors reduces oral health needs, indirectly affecting use of oral health services. This inverse correlation occurs because individuals with better socioeconomic conditions tend to demonstrate greater health literacy, greater access to healthy foods ^23^ ^,^ ^29^ ^,^ ^34^, and healthier behaviors and self-care practices, all of which contribute to better oral health conditions and, consequently, lower treatment needs ^4^ ^,^ ^23^ ^,^ ^37^ ^,^ ^38^ ^,^ ^39^. In addition to this indirect, need-mediated effect, the final models showed a direct and positive path from capacity factors to use of oral health services, indicating that the presence of predisposing and capacity factors influenced use of oral health services, even in the absence of a perceived need.

Analysis of the factor loadings of capacity factors on use of oral health services revealed weak associations for both men and women, suggesting that while economic factors and access to services have a positive impact, they are not the main determinants of use of oral health services. This corroborates Andersen’s model ^21^ ^,^ ^22^, which emphasize that the use of health services is determined by the interaction of multiple factors that can directly or indirectly impact utilization. The higher factor loading among men compared to women indicates that their use of services may be more strongly influenced by enabling conditions, such as financial resources ^1^ ^,^ ^10^ and employment status ^1^ ^,^ ^6^. A study conducted in Canada ^1^ found that low-income male workers were less likely than women in similar conditions to have visited a dentist, even after adjustment for predisposing and need factors. This may be because women generally have more positive attitudes towards dental services, better self-care behaviors, and higher oral health literacy ^6^ ^,^ ^10^ ^,^ ^11^ ^,^ ^34^. Additionally, women’s greater use of dental services may be associated with aesthetic concerns, which stem not from biological characteristics but from historically and socially constructed gender norms. These cultural pressures, which impose stricter aesthetic standards on women, may lead them to seek dental care to improve both oral health and appearance, even in the face of financial or access barriers ^1^ ^,^ ^9^ ^,^ ^40^.

Income showed the highest factor loading within capacity factors for both sexes. Many studies have demonstrated a significant association between income and use of oral health services ^3^ ^,^ ^4^ ^,^ ^5^ ^,^ ^34^ ^,^ ^39^. Individuals with higher incomes tend to have more access to private dental services, more frequent preventive consultations, and better-quality treatments ^1^ ^,^ ^3^. In contrast, those with lower income are more dependent on the public healthcare system, in which they face barriers such as long waiting times and limited service coverage ^3^ ^,^ ^34^. Other variables − such as internet access, private dental insurance, and BHU registration − also contributed to the effect of capacity factors on use of oral health services. Internet access facilitates individuals’ ability to obtain health information, schedule appointments, and even participate in remote consultations, thereby promoting service utilization ^41^. Similarly, dental insurance reduces financial barriers, promoting regular and preventive service use ^1^. BHUs are part of SUS and offer primary health care to all citizens via tax-funded public resources. Registration in BHUs is the first step for citizens to be integrated into the Brazilian health system and access health services ^3^ ^,^ ^42^.

Need factors had a positive and direct effect on use of oral health services, confirming Andersen’s theory that health needs represent the most proximal determinant of service use ^21^ ^,^ ^22^. This means that a greater need for oral care leads to a greater use of oral health services. Tooth loss and the need for prostheses are oral health conditions associated with dental caries and periodontal disease − conditions that affect the oral cavity and cause complications such as pain and infection. Thus, they require immediate professional intervention, as they affect nutrition, speech, and overall quality of life ^23^ ^,^ ^34^.

However, the factor loading for men and women had a positive but weak association between need factors and use of oral health services. This suggests that, although need factors has a direct influence on use of oral health services, the need alone is insufficient to ensure use of oral health services. Sociodemographic, economic, and service-related factors must also be considered within the broader framework of determinants of use of oral health services ^4^ ^,^ ^21^ ^,^ ^22^. The similar factor loading values between sexes suggests that the impact of need factors on use of oral health services is comparable for men and women. The slightly higher value observed among men may reflect that men are more likely to seek dental care primarily in response to acute oral health issues. For women, other factors, such as preventive behaviors and regularity of care, influence self-perceived oral health ^1^ ^,^ ^6^ ^,^ ^10^.

Tooth loss presented the highest factor loadings within need factors for both sexes, corroborating previous studies related to tooth loss and use of oral health services. These studies also report the indirect influence of social determinants in predisposing and capacity factors on inadequate use of oral health services ^1^ ^,^ ^4^ ^,^ ^39^. Financial difficulties, limited service availability, and lack of knowledge about the importance of preventive care often lead individuals to seek care only in emergencies or for pain relief ^1^ ^,^ ^34^ ^,^ ^39^, resulting in sporadic and reactive patterns of service use. In such cases, the severity of the injury or the high costs of rehabilitation frequently make tooth recovery unfeasible, leading to extractions ^34^. Consequently, tooth loss generates demand for prosthetics, which is another factor related to use of oral health services. In Brazil, primary oral healthcare mainly provides basic procedures, while specialized services, such as prosthetic treatments, remain scarce ^31^ ^,^ ^34^ ^,^ ^43^. This reality exemplifies the simultaneous interaction of predisposing, capacity, and need factors and helps explain the low factor loading of need factors in use of oral health services. Since 2004, the Brazilian National Oral Health Policy has included Dental Specialty Centers (DSCs) in the public dental care network to expand access to preventive, restorative, and rehabilitative services ^34^. However, access to DSCs for prosthetic procedures remains limited, which may explain the low factor loading of need factors in use of oral health services ^31^ ^,^ ^34^.

Self-perceived oral health presented a negative factor loading. Individuals with poor self-perceived oral health tend to have worse oral health conditions ^1^ ^,^ ^23^ ^,^ ^44^ and greater need for dental care. However, studies show that those who report poorer oral health are often the most socially and economically vulnerable, facing greater barriers to accessing oral health services ^1^ ^,^ ^4^ ^,^ ^29^ ^,^ ^39^ ^,^ ^44^. This may explain the low factor loading of the direct effect between need factors and use of oral health services.

This study has limitations, such as the use of a secondary data, individual and dichotomous variables, and the lack of temporal relationships. Dichotomization can result in loss of information by eliminating the variability inherent in the data. Contextual variables are also useful for explaining the use of health services, but they were not included due to the methodology applied. To minimize this limitation, CFA was performed using the WLSMV estimator, appropriate for categorical data, along with diagnostic tests and model fit assessments to ensure the robustness and interpretability of the final model. However, this study uses data from a national survey, enabling the exploration of complex relationships and supporting the formulation of public policies. These results reaffirm the importance of public policies for adequate use of oral health services, accounting for regional disparities and sex-specific barriers. Although the factors examined affected both sexes similarly, men were more affected, highlighting the need for targeted strategies such as direct communication approaches, workplace-based oral health programs, and the integration of oral health into men’s health initiatives.

Future mixed-methods studies are necessary to deepen the understanding of how cultural and social values influence behaviors related to use of oral health services. Such approaches would enable the development of more culturally sensitive and context-specific interventions take consider the social realities of the population.

## Data Availability

The research data are available upon request to the corresponding author.
